# Assessing Non-Invasive Liver Function in Patients with Intestinal Failure Receiving Total Parenteral Nutrition—Results from the Prospective PNLiver Trial

**DOI:** 10.3390/nu12051217

**Published:** 2020-04-26

**Authors:** Elisabeth Blüthner, Ulrich-Frank Pape, Martin Stockmann, Mirjam Karber, Sebastian Maasberg, Sophie Pevny, Undine Gerlach-Runge, Andreas Pascher, Johann Pratschke, Frank Tacke, Jan Bednarsch

**Affiliations:** 1 Department of Gastroenterology and Hepatology, Campus Virchow-Klinikum and Campus Charité Mitte, Charité-Universitätsmedizin Berlin, Charitéplatz 1, 10117 Berlin, Germany; sophie.pevny@charite.de (S.P.); frank.tacke@charite.de (F.T.); 2 Department of Surgery, Campus Virchow-Klinikum and Campus Charité Mitte, Charité-Universitätsmedizin Berlin, Augustenburger Platz 1, 13353 Berlin, Germany; martin.stockmann@charite.de (M.S.); undine.gerlach@charite.de (U.G.-R.); johann.pratschke@charite.de (J.P.); jbednarsch@ukaachen.de (J.B.); 3 Department of Internal Medicine and Gastroenterology | Asklepios Klinik St. Georg, Asklepios Medical School, Lohmühlenstr. 5, 20099 Hamburg, Germany; ul.pape@asklepios.com (U.-F.P.); s.maasberg@asklepios.com (S.M.); 4 Department of General, Visceral and Vascular Surgery, Evangelisches Krankenhaus Paul Gerhardt Stift, Paul-Gerhardt-Str. 42-45, 06886 Lutherstadt Wittenberg, Germany; 5 Berlin Institute of Health (BIH), Anna-Louisa-Karsch-Str. 2, 10178 Berlin, Germany; mirjam.karber@charite.de; 6 Department of General, Visceral and Transplantation Surgery, Münster University Hospital, Albert-Schweitzer-Campus 1, 48149 Münster, Germany; andreas.pascher@ukmuenster.de; 7 Department of General, Visceral and Transplantation Surgery, University Hospital Aachen, Rhine-Westphalia Institute of Technology, Pauwelsstraße 30, 52074 Aachen, Germany

**Keywords:** intestinal failure, intestinal failure associated liver disease, parenteral nutrition, LiMAx test, ICG test, FibroScan

## Abstract

Liver abnormalities in intestinal failure (IF) patients receiving parenteral nutrition (PN) can progress undetected by standard laboratory tests to intestinal failure associated liver disease (IFALD). The aim of this longitudinal study is to evaluate the ability of non-invasive liver function tests to assess liver function following the initiation of PN. Twenty adult patients with IF were prospectively included at PN initiation and received scheduled follow-up assessments after 6, 12, and 24 months between 2014 and 2019. Each visit included liver assessment (LiMAx [Liver Maximum Capacity] test, ICG [indocyanine green] test, FibroScan), laboratory tests (standard laboratory test, NAFLD [non-alcoholic fatty liver disease] score, FIB–4 [fibrosis-4] score), nutritional status (bioelectrical impedance analysis, indirect calorimetry), and quality of life assessment. The patients were categorized post-hoc based on their continuous need for PN into a reduced parenteral nutrition (RPN) group and a stable parenteral nutrition (SPN) group. While the SPN group (*n* = 9) had significantly shorter small bowel length and poorer nutritional status at baseline compared to the RPN group (*n* = 11), no difference in liver function was observed between the distinct groups. Over time, liver function determined by LiMAx did continuously decrease from baseline to 24 months in the SPN group but remained stable in the RPN group. This decrease in liver function assessed with LiMAx in the SPN group preceded deterioration of all other investigated liver function tests during the study period. Our results suggest that the liver function over time is primarily determined by the degree of intestinal failure. Furthermore, the LiMAx test appeared more sensitive in detecting early changes in liver function in comparison to other liver function tests.

## 1. Introduction

Intestinal failure (IF) is a malabsorptive disorder resulting from the physical loss of small bowel mass and/or functionality [1]. Although there are treatments such as the potentially curative intestinal transplantation and medical treatment with glucagon-like peptide-2 (GLP–2) analogues, which aim to maximize absorption in the remnant bowel, parenteral nutrition (PN) remains the standard of care in the majority of patients [2,3]. Despite PN’s ability to dramatically extend life expectancy for patients with IF, patients on prolonged PN are at risk for a spectrum of hepatobiliary disorders, ranging from steatosis to intestinal failure associated liver disease (IFALD). IFALD is one of the most important factors limiting long-term survival of patients with IF [4]. Despite this, our knowledge of the pathogenesis and the time-window to appropriately treat hepatobiliary complications remains incomplete [5]. Routine laboratory testing is recommended to monitor liver function. An estimated 19%–95% of patients receiving PN have abnormal liver function tests, however, the correlations between these elevated liver enzyme levels and the actual degree of histologic injury is poor. Therefore, the European Society for Parenteral and Enteral Nutrition (ESPEN) guideline advocates the use of serial liver biopsies as the gold standard for detecting IFALD [1,6]. Despite these recommendations, both the acceptance and accuracy of liver biopsies for this purpose are limited by their invasiveness and intra- and interobserver variability [7]. Patients receiving PN would therefore benefit considerably from a non-invasive assessment for disease progression to IFALD.

Our group has previously evaluated the capability of the ^13^C-based breath test LiMAx (Liver Maximum Capacity) in diagnosing IFALD in a large cross-sectional cohort receiving long-term PN [8]. In the present study, we aim to expand this previous research by comparing the effectiveness of non-invasive liver function tests with standard liver function tests for examining liver function and detecting hepatic dysfunction over time in patients receiving PN.

## 2. Material and Methods

### 2.1. Study Concept

Between 2014 and 2019, we conducted a prospective 24-month follow-up study for patients with IF presenting to our multidisciplinary intestinal failure team at Charité Universitätsmedizin Berlin. In total, we enrolled 20 adult patients with IF receiving PN for less than three months who required at least three intravenous nutrition bags per week at baseline. Exclusion criteria were underlying liver disease unrelated to PN and presence of active cancer. Baseline was defined as the first assessment after written informed consent was obtained. Follow-up visits were conducted at 6, 12, and 24 months and included clinical examinations, liver assessments, comprehensive blood tests, quality of life, and nutritional status assessments after a minimum of six hours of fasting. Patients’ current nutrition regimens were documented and any complications they experienced were recorded. 

Patients were categorized post-hoc into the reduced parenteral nutrition (RPN) group if their mean interval reduction in parenteral calories was 20% or greater. Patients displaying a mean interval reduction in parenteral calories less than 20% were assigned to the stable parenteral nutrition (SPN) group.

The institutional ethics committee approved the study protocol (EA1/110/14), which conforms to the STROBE statement and is registered as DRKS00010993 [9]. The study complied with the ethical standards of the 1964 Declaration of Helsinki and its last revision of 2013.

### 2.2. Clinical Management

Parenteral nutrition was prescribed based on patients’ nutritional status, laboratory tests, digestive anatomy, and in accordance with the national and international clinical practice guidelines [1,10]. Remnant small bowel length and gastrointestinal anatomy were taken from either radiological follow-through examinations (FTE) or surgical reports. Digestive anatomy was categorized according to the criteria published by Messing et al. into type I (end-enterostomy), type II (jejunocolic anastomosis), and type III (jejunoileocolic anastomosis) [11]. Serum citrulline level was recorded as a marker of absorptive enterocyte mass [12].

### 2.3. Liver Assessment

Liver assessment was based on laboratory tests, dynamic liver function tests, and liver stiffness measurements. A fasting blood sample was drawn to determine standard liver functions tests (LFT) consisting of levels of bilirubin, aspartate aminotransferase (AST), alanine aminotransferase (ALT), alkaline phosphatase (AP), and gamma-glutamyltransferase (GGT). The following additional laboratory tests were also collected: glutamate dehydrogenase (GLDH), cholinesterase, ammonia, albumin, blood count, prothrombin time, international normalized ratio (INR), and clotting factors II and VII. We also calculated the non-alcoholic fatty liver disease (NAFLD) and fibrosis-4 score (FIB-4) to predict fibrosis as described elsewhere [13,14].

Liver Maximum Capacity (LiMAx) reflects cytochrome P450 1A2 activity and is therefore recognized as a reliable marker of liver function capacity. The patient is placed in a resting horizontal position and breathes into a respiratory mask that is connected to a detection device that continuously measures the ^13^CO_2_/^12^CO_2_ ratio. During the test, a bodyweight-adjusted solution of ^13^C-labeled methacetin is administered intravenously. The ^13^C-labeled methacetin is instantaneously metabolized into paracetamol and ^13^CO_2_, which is then exhaled. The amount of exhaled ^13^CO_2_ is proportional to the patient’s liver function capacity [15]. LiMAx values >315 μg/kg/h are considered normal [16]. 

The indocyanine green (ICG) test reflects indocyanine green elimination capacity over a short time frame. The ICG test involves a bodyweight-adjusted intravenous injection of indocyanine that is then taken up by hepatocytes and subsequently eliminated into the bile. The plasma ICG disappearance rate was measured using a special device (Dye Densitogram Analyzer DDG2001, Nihon Khoden, Tokyo, Japan). ICG values over 18%/min are considered normal [17].

Liver stiffness measurements were carried out using the ultrasonographic FibroScan technique (Echosens, Paris, France), which makes use of the transient elastography principle. The examination was performed with the patients in supine position with their right arm in maximal abduction. Liver stiffness was examined by placing the M-mode transducer above the right lobe of the liver and scanning through the intercostal space as previously described by Sandrin et al. [18]. FibroScan results are expressed as the median stiffness value in kilopascals (kPa). Measurements with ten successful acquisitions and a success rate ≥60% were considered reliable.

### 2.4. Nutritional Status Assessment

Energy requirements were measured by indirect calorimetry (Quark RMR, COSMED, Rome, Italy) as described elsewhere [19]. Individual body composition was evaluated by a bioelectrical impedance device (Nutriguard-M, Data Input, Pöcking, Germany) as previously described [20].

### 2.5. Quality of Life Assessment

Quality of life (QoL) for each patient was evaluated with two different questionnaires. Short Form 36 (SF-36) is the most commonly used non-disease-specific instrument in registered clinical trials today and has been validated for German language users. The SF-36 examines QoL in eight domains, scoring each between 0 and 100, and two summary scales. The scores were calculated using Hogrefe Testsystem 5 (HTS 5) (Hogrefe, Göttingen, Germany) with higher scores indicating higher levels of QoL [21]. The Short Bowel Syndrome-Quality of Life (SBS-QoL) questionnaire is a disease-specific instrument evaluating different aspects of QoL on a visual analogue scale. Seventeen items are collated into two subscales, with a total summary scale ranging from 0–170. Higher scores represent lower levels of QoL [22].

### 2.6. Statistical Analysis

All statistical analyses were performed with SPSS Statistics 25 software (SPSS Inc., Chicago, IL, USA). Figures were created using Prism 6 software (GraphPad Software, Inc., La Jolla, CA, USA). Continuous variables are reported as median and interquartile range, and categorical data are reported as numbers and percentages if not noted otherwise. Differences between the groups were compared using the Mann–Whitney U test or Kruskal–Wallis test as appropriate for the size and scale of the given variable. Paired data were analyzed using the Wilcoxon rank sum test. Statistical significance was set at the 5% level.

## 3. Results

### 3.1. Baseline Characteristics

After screening of 90 patients presenting in our department between 2014 and 2017, a total of 20 patients fulfilled all inclusion and exclusion criteria. These patients were prospectively included and followed up at 6, 12, and 24 months. The median age at enrolment was 58 (38–70) years and the median PN duration was 2.0 (0.3–3.0) months. Mesenteric ischemia was the most frequent cause of IF (35%), followed by obstructive ileus secondary to adhesions (30%), and inflammatory bowel disease (20%). The median number of infusions per week was 7.0 (5.5–7.0). According to Messing’s anatomic criteria, eight patients (40%) were classified as type I, seven patients (35%) were type II, and five patients (25%) were type III. PN was required in 14 patients (70%) after one year and in eight patients (40%) after two years. The number of patients who were eligible for analysis were nine, nine, seven, and five patients in the SPN group and 11, 11, seven, and three patients in the RPN at the 6, 12, and 24 months visit, respectively. A synopsis of the baseline characteristics is presented in detail in Table 1. 

### 3.2. Quality of Life

**Figure 1:**
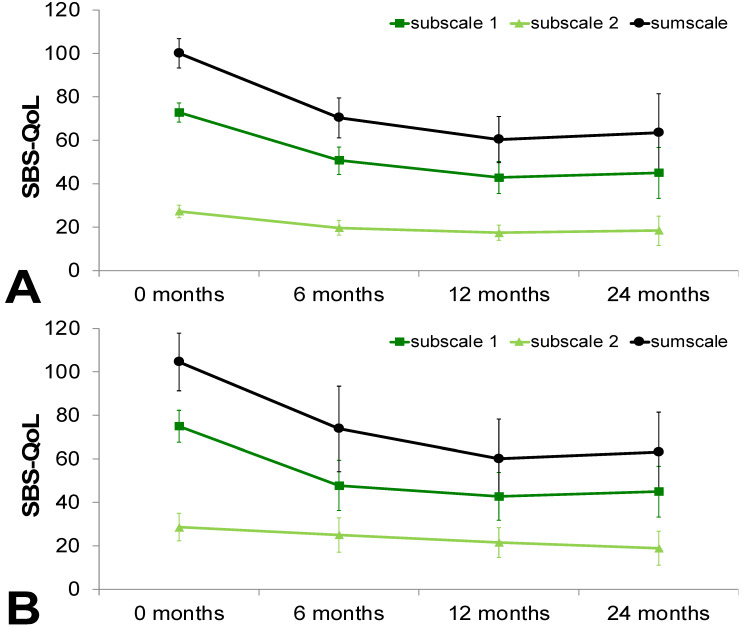
Quality of life as determined by the SBS–QoL. Quality of life improved within the study period in total cohort (**A**) and a subgroup excluding patients who were weaned off parenteral nutrition (**B**). Data presented as mean ± standard deviation. SBS-QoL, Short Bowel Syndrome-Quality of Life.

The patients experienced significant improvements in quality of life as determined by the SBS-QoL sum score when comparing baseline and 6 month scores (103 (72–127) vs. 62 (40–96); *p* = 0.003) as well as baseline and 12 months (103 (72–127) vs. 65 (23–81); *p* = 0.004). The sum score also improved between the 12 and 24 month time points (65 (23–81), vs. 43 (35–88), *p* = 0.612; Figure 1A). A subgroup excluding patients who were weaned off PN (*n* = 12) during the study period showed similar results as shown in Figure 1B.

**Figure 2:**
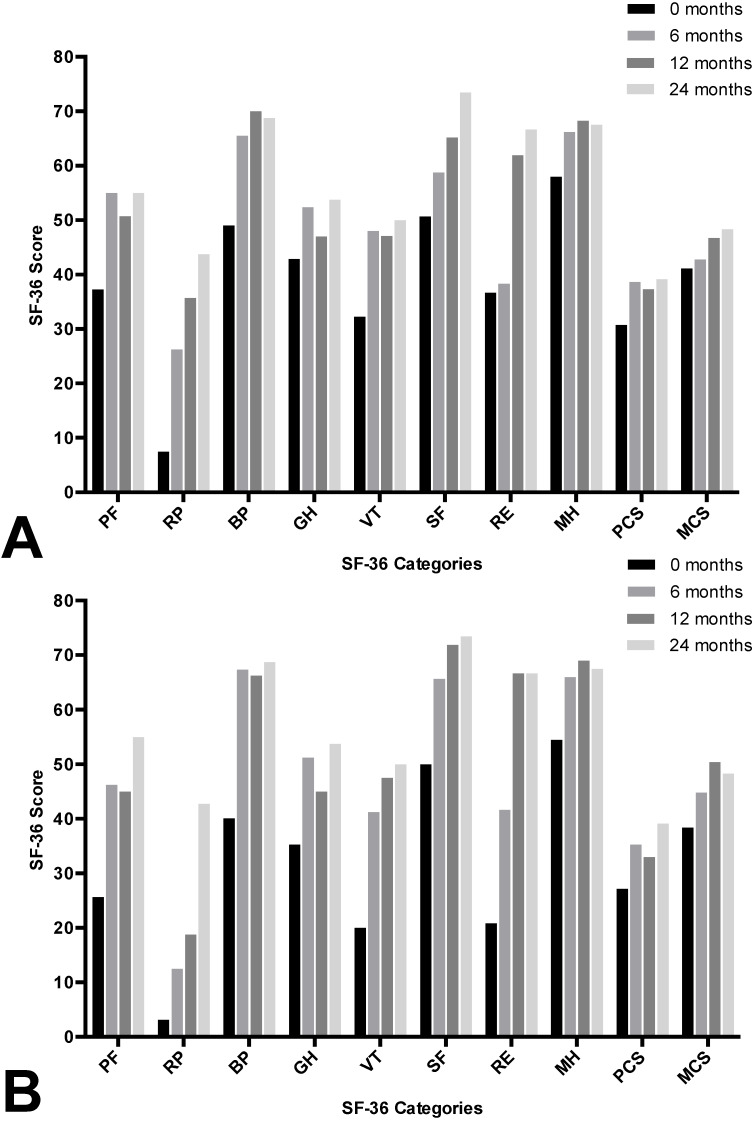
Quality of life determined by the SF–36. Quality of life improves in eight domains and in two summary scales for the total cohort (**A**) and in a subgroup excluding patients who were weaned off PN (**B**). Data presented as mean ± standard deviation. PF, Physical Functioning; RP, Role-physical; BP, Bodily Pain; GH, General Health; VT, Vitality; SF, Social Functioning; SF-36, Short Form 36; RE, Role-emotional; MH, Mental Health; PCS, Physical Component Summary; MCS, Mental Component Summary.

The SF-36 Physical Component Summary (PCS) showed a significant improvement in quality of life from baseline to 6 months (29 (24–33) vs. 39 (31–46); 0.002), stayed constant between the 6 and 12 month time points (39 (31–46) vs. 36 (30–46); *p* = 0.069) and slightly improved between 12 and 24 months (36 (30–46) vs. 40 (30–49); *p* = 0.093). The Mental Component Summary (MCS) improved slightly from enrolment (42 (31–51)) to 6 months (43 (38–51)), 12 months (51 (34–60)), and 24 months (54 (38–58)) as seen in Figure 2A. The subgroup cohort is separately illustrated in Figure 2B demonstrating comparable results.

### 3.3. Comparison between Reduced vs. Stable Parenteral Nutrition Group at Baseline

A comparative analysis including nine patients with reduced parenteral nutrition (RPN) and 11 patients with stable parenteral nutrition (SPN) was conducted. No significant differences with respect to demographic data or baseline liver assessments were observed. However, at enrolment, the SPN group showed poor nutritional status with significantly reduced phase angle (PhA) (4.0 (3.3–4.3) vs. 4.5 (4.1–5.4); *p* = 0.046), body cell mass (BCM; 17.8 (12.5–20.3) vs. 21.7 (19.8–28.8) kg; *p* = 0.025), and calculated resting energy expenditure (REE; 1180 (1010–1255) vs. 1300 (1240–1530) kcal; *p* = 0.025) as determined by bioelectrical impedance analysis (BIA). Accordingly, body mass index (BMI) was slightly lower in the SPN group than in the RPN group (18.8 (15.8–22.1) vs. 21.5 (20.5–26.3) kg/m^2^; *p* = 0.080) but did not show statistical significance in our analysis. Total PN per infusion was significantly decreased in the SPN group (1240 (1100–1505) vs. 1600 (1435–1600) kcal; *p* = 0.046) as well as lipids per infusion (50 (39–58) vs. 60 (56–76) g; *p* = 0.031). Furthermore, the remnant small bowel length was significantly lower in the SPN group in comparison to the patients in the RPN group (94 (60–112) vs. 117 (104–144) cm; *p* = 0.046). The baseline values of the groups are presented in Table 1 and Table 2.

### 3.4. Longitudinal Assessment

Longitudinal differences between the two distinct groups regarding their nutritional status und parenteral nutrition program are presented in Table 2.

In the RPN group, infusions per week decreased more than half between enrolment (7.0 (5.0–7.0)) and 24 months (2.0 (2.0–)). In the SPN group infusions per week remained constant between enrolment (7.0 (5.5–7.0)) and 24 months (7.0 (4.5–7.0)). Despite the PN reduction in the RPN group, BMI remained unaltered over the study period from baseline (21.5 (20.5–26.3) kg/m^2^) to 24 months (22.5 (19.8–) kg/m^2^). Conversely, due to stable parenteral support, the SPN group showed a BMI increase from baseline (18.8 (15.8–22.1) kg/m^2^) to 24 months (21.4 (17.2–23.6) kg/m^2^). Accordingly, the nutritional status determined by BIA was stable in the RPN group with unaltered BCM and PhA from baseline to 24 months. At the 6 month mark however, there was a significant difference (*p* = 0.005) between the SPN and RPN groups’ BCM changes at baseline (17.8 (12.5–20.3) vs. 21.7 (19.8–28.8) kg) and at 6 months (22.9 (18.4–26.2) vs. 24.8 (19.8–30.8) kg), respectively. Similarly, the PhA remained unaltered in the RPN group while in the SPN group there was a significant increase within the study period (*p* = 0.026) from baseline (4.0 (3.3–4.3) vs. 4.5 (4.1–5.4)°) to 6 months (5.3 (4.5–6.5) vs. 4.8 (4.5–5.5)°) between the groups. 

### 3.5. Liver Assessment

The LiMAx test was positively significantly correlated with the ICG test (*p* = 0.008) and negatively significantly correlated the FibroScan at baseline (*p* = 0.011) in all patients (data not shown). The LiMAx values in the SPN group decreased between each time interval from baseline (580 (411–671) µg/kg/h), to 6 months (433 (335–532) µg/kg/h), to 12 months (439 (287–455) µg/kg/h), and 24 months (328 (251–370) µg/kg/h). In the RPN group, the LiMAx values increased from baseline (470 (350–569) µg/kg/h), to 6 months (603 (398–786) µg/kg/h), and 12 months (610 (515–767) µg/kg/h). After 24 months, eight out of 11 patients in the RPN group were weaned off PN and subsequently excluded from further analysis. The LiMAx test was significantly different between the groups at 12 months (*p* = 0.038). There was a significant difference in mean LiMAx change from baseline to 6 months (*p* = 0.031), and from baseline to 12 months (*p* = 0.011) between the groups.

ICG values decreased in both the SPN and RPN groups from baseline (25.4 (18.7–29.5) vs. 20.3 (15.6–23.9) %/min) to 24 months (16.9 (9.7–20.4) vs. 17.1 (5.1–) %/min). There was no statistical difference between the groups throughout the follow-up assessments. The FibroScan values remained unaltered within the first 12 months, but increased slightly from 12 months (5.7 [4.3–8.8] vs. 6.6 (4.3–9.6) kPa) to 24 months (6.8 (4.2–18.2) vs. 12.5 (5.7–) kPa) for the SPN and RPN groups, respectively. This difference did not however achieve statistical significance due to high variability.

**Figure 3:**
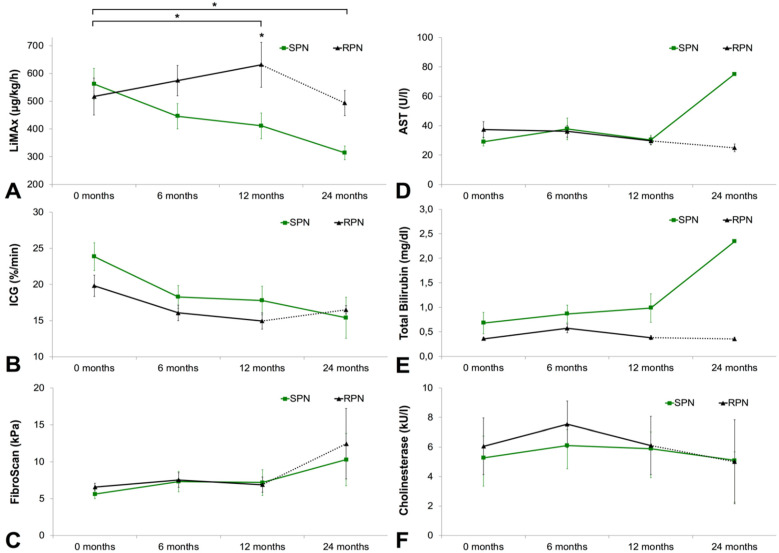
Liver function over time as determined by liver function tests. (**A**) LiMAx test; (**B**) ICG test; (**C**) FibroScan; (**D**) AST; (**E**) Total Bilirubin; (**F**) Cholinesterase. Data presented as mean ± standard deviation. RPN group presented in dashed lines between the 12- and 24-month time points due to reduced sample size as a consequence of PN weaning. *, *p* < 0.05; AST, aspartate aminotransferase; LiMAx, maximum liver function capacity; ICG, indocyanine green; RPN, reduced parenteral nutrition; SPN, stable parenteral nutrition.

Transaminases slightly decreased over time in the RPN group, whereas in the SPN group transaminases remained stable from baseline to 12 months, and subsequently increased between 12 months to 24 months (see Table 2). In line with these results, serum bilirubin remained stable in the RPN group and increased between 12 months (0.71 (0.24–1.55) mg/dL) and 24 months (0.91 (0.30–5.1) mg/dL) in the SPN group. Surrogate markers of synthetic liver function (e.g., cholinesterase) and fibrosis scores were unaffected over time. Detailed analyses of liver function over time by LiMAx, ICG, FibroScan, laboratory testing, and laboratory scores are presented in Figure 3 and Table 2.

## 4. Discussion

We prospectively enrolled 20 patients after PN initiation and assessed their liver function systematically over the course of two years. Due to impaired nutritional status and short remnant bowel length, nine patients were dependent on total parenteral nutrition (SPN group), whereas 11 patients were able to halve their parenteral caloric intake over time (RPN group). This prospective study has shown that liver function over time is determined by the severity of IF, as defined by remnant small bowel length and the need for parenteral support over time. Dynamic liver function assessment by LiMAx test was more sensitive in detecting early changes in liver function when compared to the ICG test, FibroScan, and standard laboratory testing. Despite a different course of liver function, quality of life determined by SBS-QoL and SF-36 improved during study period in both groups.

Since parenteral nutrition was introduced in the late 1940s, IF patient survival and quality of life have markedly improved. This reflects advances in PN mixtures, medical treatments, and patient-centred multidisciplinary care [23]. However, progressive liver disease and the loss of central venous access are limiting factors for long-term survival and are still leading indications for small bowel transplantation in patients with IF [24]. The ability to detect early liver dysfunction is vitally important for appropriately timing specialist referrals and determining the correctly indicated transplantation, as higher mortality is seen in intestine–liver transplantations than in intestine-alone transplantations [25,26]. With the exception of hepatic cirrhosis, liver fibrosis is reversible after patients are weaned off PN or undergo intestinal transplantation [27]. Therefore, establishing the non-invasive ability to accurately detect the progression from hepatic fibrosis to irreversible cirrhosis would marks a significant advance in clinical management. Potential therapeutic approaches are the administration of PN enriched with omega-3 fatty acids (which have been shown to diminish hepatic steatosis as a result of their anti-inflammatory effects), and the application of GLP-2 analogues or chyme reinfusions (which increase intestinal absorption and thereby lead to reduced reliance on parenteral support [2,28,29]). Nevertheless, identifying patients at heightened risk for IFALD still presents a considerable challenge.

Abnormal LFTs are commonly seen in parenterally nourished patients, but it has been observed that some patients progress to liver cirrhosis in the setting of normal enzyme levels [30]. Interestingly, Naini et al. have previously shown that biochemical enzymes do not correlate with the degree of fibrosis seen histologically [6]. This may explain how laboratory-based scores, such as aspartate aminotransferase to platelet ratio index (APRI) and Fibrosis-4 (FIB-4) scores, are significantly correlated with the severity of histological cholestasis but fail to show a correlation with stage of fibrosis [31]. The FibroScan was introduced to overcome these limitations as a non-invasive alternative. However, it was later demonstrated that the FibroScan did not correlate with histological fibrosis, but rather with histological cholestasis in adult patients receiving PN [31]. In accordance with these results, we could not demonstrate a significant progress of fibrosis by NAFLD, FIB-4 score, and FibroScan in both groups (Table 2). Notably, the FibroScan and APRI score have shown promising results in pediatric patients [32]. This observation may reflect the fact that children are particularly prone to developing cholesteric liver disease, whereas steatosis is more commonly seen in adult patients [33]. Interestingly, Alizai et al. demonstrated in morbidly obese patients receiving bariatric surgery that the BMI loss correlated with LiMAx improvements. Preoperatively, NAFLD activity score determined by liver biopsy correlated negatively with liver function capacity suggesting that alterations of BMI may also change liver function histologic improvements of non-alcoholic steatohepatitis (NASH) [34]. The novel FibroScan vibration-controlled transient elastography controlled attenuation parameter (CAP) have shown promising results to assess liver steatosis [35]. However, a small prospective study including 25 intestinal failure patients could not demonstrated a significant dynamic of steatosis and fibrosis by FibroScan stiffness and CAP parameter [36]. It is assumed that IFALD refers to liver injury as a result of several factors relating to several histological abnormalities as previously above. Currently, further investigation are needed to clarify this pathomechanism of IFALD [35].

In accordance with these limitations of laboratory parameters, we did not observe significant differences of standard liver function tests between or within the RPN and SPN groups, respectively. Moreover, although the difference of bilirubin between the RPN and SPN group after 24 months was notable, it did not achieve the level of significance (*p* = 0.209) compared to LiMAx which was significantly different between the groups after 12 months (*p* = 0.038). Also, the changes of LiMAx values within the groups were statistically significant compared to changes of bilirubin (see Table 2) Despite a lack of statistical significance regarding bilirubin, this observation leaves room for speculations. A possible explanation might be that changes in LiMAx precede alterations of standard liver function tests as indicated by the continuous increase and decrease of LiMAx in the RPN and the SPN group, respectively. Nevertheless, it has also to be acknowledged that the results at 24 months have to be interpreted taking a notable number of patients who were weaned off PN into consideration, which reduces the validity of this particular time point.

Previously published data from our group suggests that the LiMAx test may be a reliable non-invasive method for detecting hepatic dysfunction in IF patients [8]. The LiMAx test has been successfully evaluated as a new diagnostic test in various clinical scenarios. At present, LiMAx is routinely used prior to hepatectomy for predicting postoperative liver failure [15]. The LiMAx test is also a sensitive tool for the early detection of both sepsis-related hepatic dysfunction and graft dysfunction following liver transplantation [37,38]. In summary, the LiMAx has shown comparable and perhaps higher prognostic value for predicting IFALD progression than conventionally used laboratory tests, scoring systems, or imaging-based liver function tests [39,40].

Due to its multifactorial etiology, the umbrella term IFALD has broadly replaced the use of PN-associated liver disease (PNALD) to emphasize the importance of both nutrition-related and patient-related risk factors for hepatic damage [25]. A study by Luman et al. has shown that reduced small bowel length and high parenteral caloric intake are the two major risk factors for IFALD [41]. Separating the effect sizes of these two risk factors is challenging as the amount of PN is dictated by the severity of IF. In the present study, we grouped patients according to their need for PN over time and found that this factor significantly explained variability in their liver function over time as measured by LiMAx (Figure 3). The patients in the SPN group were dependent on long-term PN due to their poor nutritional status at enrolment, as determined by BMI and BIA measurements. Despite essentially halving the caloric intake in the RPN group over two years, their nutritional status was comparable to the SPN group. Citrulline has been extensively validated as a powerful marker of small bowel absorptive capacity [12,42]. Surprisingly, the RPN group had lower citrulline levels at PN initiation, however their citrulline levels also increased markedly faster than in the SPN group (Table 2). However, it has to be noted that patients that were weaned from PN during the study period were not included in the later study visits. Therefore, the citrulline progress may be biased and must be interpreted with caution. From a clinical point of view, it may be more useful to trend the progression of citrulline levels rather than taking cut-off values into account. In summary, we have demonstrated in this subgroup analysis that higher degrees of malabsorption correspond to higher risk of IFALD.

Assessments of quality of life as measured by the SBS-QoL and SF-36 has shown comparable results (see Figure 1 and Figure 2). There was a constant improvement in quality of life within in study period of 24 months. In line with these results, previous studies have demonstrated lower quality of life in patients on short-term PN in comparison to patients on long-term PN, with these differences remaining stable over time [43,44]. However, it is still unclear whether QoL improves due to disease rehabilitation or due to adaptation to PN since in this study, follow-up assessments were carried out only on patients on PN. 

There are several limitations to our study. Firstly, liver biopsy as the current gold-standard for diagnosing IFALD was not part of the study protocol due to ethical considerations. Subsequently, our results and claims have to be interpreted with caution and warrant further validations by other groups. However, LiMAx has been shown to correlate well with histological changes in different stages of liver fibrosis and cirrhosis in chronic liver disease [45]. Despite being certainly different in pathogenesis to other more common causes of chronic liver disease, it is assumable that LiMAx might also be associated with the histological patterns of IFALD. Secondly, despite its prospective design, this observational study is limited by the small sample size. Furthermore, a number of patients were able to cease PN usage after one to two years. Therefore, follow-up data are only available in eight patients at the two-year time point. Epidemiological data has shown that PN-dependence probabilities range from 74%–82% and 64%–80% at one and two years, respectively [11,46,47]. In this study, PN-dependence was slightly lower. Of all 20 patients, 14 individuals (70%) were PN-dependent at one-year follow-up while eight individuals (40%) were PN-dependent at two-year follow-up. However, we do not consider this to be a weakness of our study because the primary focus was on assessing liver function in parenteral nourished patients. Finally, a larger multicenter study is needed to validate our findings and to define individual cut-off values for IFALD progression, which would prove highly useful for clinical practice.

## 5. Conclusions

In conclusion, we have shown that liver function over time is determined by the severity of intestinal failure, as demonstrated by both undiminished need for parenteral support and amount of remnant small bowel. After taking the aforementioned limitations into consideration, our study indicates that the LiMAx was more sensitive in detecting early changes in liver function as compared to ICG test, FibroScan, and standard laboratory tests. Our results also suggest that routinely applied LiMAx testing may be a useful tool for detection of hepatic dysfunction in patients receiving PN before it becomes clinically apparent.

## Figures and Tables

**Table 1:** Baseline characteristics.

Descriptive Data	Total Cohort	SPN (*n* = 9)	RPN (*n* = 11)	*p*-Value
Age, years	58 (38–70)	50 (27–62)	68 (57–73)	0.112
Sex, f/m	10/10	6/3	4/7	0.178
BMI, kg/m^2^	21.5 (18.5–22.3)	18.8 (15.8–22.1)	21.5 (20.5–26.3)	0.080
Primary disease, *n* (%)				0.246
Mesenteric ischemia	7 (35.0)	3 (33.3)	4 (36.4)	
Obstructive ileus	6 (30.0)	1 (11.1)	5 (45.5)	
Inflammatory bowel disease	4 (20.0)	3 (33.3)	1 (9.1)	
Post-surgical complications	1 (5.0)	0 (0)	1 (9.1)	
Abdominal trauma	1 (5.0)	1 (11.1)	0 (0)	
Benign abdominal tumor	1 (5.0)	1 (11.1)	0 (0)	
**Bioelectrical Impedance Analysis**				
Body cell mass, kg	20.1 (15.2–23.7)	17.8 (12.5–20.3)	21.7 (19.8–28.8)	**0.025**
Phase angle, °	3.7 (4.3–4.9)	4.0 (3.3–4.3)	4.5 (4.1–5.4)	**0.046**
Calculated REE, kcal	1250 (1098–1360)	1180 (1010–1255)	1300 (1240–1530)	**0.025**
**Calorimetry**				
CO_2_ volume, L/min	182 (149–212)	168 (128–208)	182 (149–227)	0.545
O_2_ volume, L/min	209 (192–255)	199 (187–250)	213 (202–280)	0.272
Measured REE, kcal	1440 (1303–1764)	1372 (1247–1732)	1440 (1371–1927)	0.310
**Parenteral Nutrition Program**				
Oral intake, n (%)	19 (95)	8 (88.9)	11 (100)	0.257
Duration of PN, months	2.0 (0.3–3.0)	1.0 (0–2.5)	3.0 (1.0–3.0)	0.131
Infusions per week, n	7.0 (5.5–7.0)	7.0 (5.5–7.0)	7.0 (5.0–7.0)	0.941
Total PN, kcal/infusion	1518 (1240–1600)	1240 (1100–1505)	1600 (1435–1600)	**0.046**
Total PN, kcal/week	9485 (7175–11,200)	8680 (5980–10,535)	11,200 (8000–11,200)	0.230
Amino acid, g/infusion	68 (56–85)	60 (50–78)	85 (60–85)	0.131
Glucose, g/infusion	140 (125–165)	135 (125–158)	155 (125–165)	0.456
Lipids, g/infusion	58 (43–74)	50 (39–58)	60 (56–76)	**0.031**
Soybean oil, g/infusion	16 (12–22)	15 (12–17)	17 (12–24)	0.766
Olive oil, g/infusion	14 (10–36)	10 (9–14)	20 (14–48)	**0.046**
MCT, g/infusion	14 (3–22)	12 (11–16)	17 (0–24)	0.882
Fish oil, g/infusion	6 (0–8)	6 (3–8)	6 (0–11)	0.882
**Anatomy**				
Length of remaining small intestine, cm	104 (75–132)	94 (60–112)	117 (104–144)	**0.046**
Length of remaining small-intestine, n (%)				**0.031**
≤50 cm	1 (5)	1 (11)	0 (0)	
51–100 cm	6 (30)	5 (56)	1 (9)	
>100 cm	10 (50)	3 (33)	7 (64)	
Unknown	3 (15)	0 (0)	3 (27)	
Digestive anatomy groups, n (%)				0.930
Type I	8 (40)	4 (44)	4 (36)	
Type II	7 (35)	3 (33)	4 (36)	
Type III	5 (25)	2 (22)	3 (27)	
Presence of intestinal fistula, n (%)	2 (10)	1 (11)	1 (9)	0.881
Presence of ileo-cecal valve, n (%)	2 (10)	0 (0)	2 (18)	0.178
Stoma, n (%)	9 (45)	5 (56)	4 (36)	0.391
Colon anatomy, n (%)				0.611
Intact colon	2 (10)	1 (11)	1 (9)	
(Extended) colectomy	9 (45)	3 (33)	6 (55)	
Subtotal colectomy	1 (5)	1 (11)	0 (0)	
Total Colectomy	8 (40)	4 (44)	4 (36)	
**Liver Assessment**				
LiMAx, µg/kg/h	483 (374–639)	580 (411–671)	470 (350–569)	0.370
ICG, %/min	21.5 (16.7–26.6)	25.4 (18.7–29.5)	20.3 (15.6–23.9)	0.131
FibroScan, kPa	5.6 (4.5–7.3)	4.9 (4.1–7.1)	6.8 (5.0–7.8)	0.200
**Complications**				
CRBSI, n	4 (20)	2 (22.2)	2 (18.2)	0.822
**Laboratory Tests**				
NAFLD	−2.65 (−4.53–−1.22)	−3.62 (−5.34–−1.96)	−1.74 (−2.91–−0.78)	0.095
FIB-4	1.02 (0.53–1.89)	0.53 (0.35–1.38)	1.51 (0.72–2.10)	**0.038**
AST, U/L	32 (23–42)	30 (21–36)	37 (24–50)	0.261
ALT, U/L	37 (24–74)	36 (22–65)	41 (28–104)	0.412
AP, U/L	107 (78–156)	108 (86–378)	98 (71–150)	0.456
GGT, U/L	110 (37–286)	191 (41–472)	100 (31–194)	0.503
Total bilirubin, mg/dL	0.40 (0.26–0.47)	0.44 (0.26–1.00)	0.39 (0.24–0.44)	0.412
Conjugated bilirubin, mg/dL	0.22 (0.18–0.32)	0.26 (0.18–0.51)	0.21 (0.18–0.23)	0.370
Serum albumin, g/l	37 (33–41)	36 (32–39)	37 (33–43)	0.370
Cholinesterase, kU/L	5.6 (4.1–6.7)	5.6 (4.0–6.4)	5.7 (4.0–6.9)	0.766
Factor II, %	83 (74–101)	83 (73–88)	97 (73–106)	0.331
Factor VII, %	118 (99–145)	117 (93–139)	122 (100–187)	0.603
INR	1.1 (1.0–1.2)	1.1 (1.0–1.1)	1.0 (1.0–1.3)	0.824
Creatinine, mg/dL	0.9 (0.6–1.1)	0.8 (0.5–1.0)	0.9 (0.7–1.1)	0.331
CRP, mg/L	0.5 (0.2–1.5)	0.4 (0.1–1.9)	0.7 (0.2–1.4)	0.824
WBC, /nL	6.7 (5.5–9.0)	5.8 (4.9–8.8)	6.9 (6.0–9.0)	0.175
Citrulline, µmol/L	22 (16–29)	25 (15–41)	21 (16–23)	0.503

Bold values indicate *p* < 0.05, ALT, alanine aminotransferase; AP, alkaline phosphatase; AST, aspartate aminotransferase; BMI, body mass index; CRBSI, catheter related blood stream infection; CRP, C-reactive protein; FIB-4, firbrosis-4 score; GGT, gamma-glutamyltransferase; ICG, indocyanine green; INR; international normalized ratio; LiMAx, maximum liver function capacity; MCT, medium-chain triglycerides; NAFLD, non-alcoholic fatty liver disease; PN, parenteral nutrition; REE, REE, resting energy expenditure; RPN, reduced parenteral nutrition; SPN, stable parenteral nutrition; WBC, White blood cell.

**Table 2:** Longitudinal assessment.

Parameter	Baseline	6 Months	12 Months	24 Months
**Number of patients, *n* (%)**				
SPN	9 (100)	9 (100)	7 (78)	5 (56)
RPN	11 (100)	11 (100)	7 (64)	3 (27)
**BMI, kg/m^2^**			#, Ω	
SPN	18.8 (15.8–22.1)	21.7 (18.1–23.6)	23.2 (18.0–26.8)	21.4 (17.2–23.6)
RPN	21.5 (20.5–26.3)	23.5 (20.3–25.1)	23.2 (20.1–26.7)	22.5 (19.8–)
**Body cell mass, kg**	*	#		
SPN	17.8 (12.5–20.3)	22.9 (18.4–26.2)	20.6 (15.7–26.8)	20.1 (16.1–31.5)
RPN	21.7 (19.8–28.8)	24.8 (19.8–30.8)	21.7 (17.8–24.9)	23.9 (19.3–)
**Phase angle, °**	*	#		
SPN	4.0 (3.3–4.3)	5.3 (4.5–6.5)	4.7 (4.1–6.0)	4.8 (4.0–5.6)
RPN	4.5 (4.1–5.4)	4.8 (4.5 – 5.5)	4.3 (3.5 – 4.7)	5.2 (3.9–)
**Calculated REE, kcal**	*			
SPN	1180 (1010–1255)	1340 (1195–1440)	1270 (1010–1460)	1250 (1125–1615)
RPN	1300 (1240–1530)	1400 (1240–1590)	1300 (1180–1400)	1370 (1230–)
**Infusions per week, *n***		#		
SPN	7.0 (5.5–7.0)	7.0 (4.0–7.0)	7.0 (2.0–7.0)	7.0 (4.5–7.0)
RPN	7.0 (5.0–7.0)	4.0 (2.0–5.0)	4.0 (2.0–7.0)	2.0 (2.0–)
**Total PN, kcal/infusion**	*		Ω	
SPN	1240 (1100–1505)	1410 (1302–1506)	1560 (1400–1665)	1400 (1338–1830)
RPN	1600 (1435–1600)	1600 (1390–1600)	1440 (1390–1600)	1440 (1300–)
**Total PN, kcal/week**		#		
SPN	8680 (5980–10,535)	8460 (5502–9982)	9800 (3330–11,550)	9450 (6993–10,675)
RPN	11,200 (8000–11,200)	6080 (2200–7200)	5200 (3200–10,045)	3200 (2880–)
**Amino acid, g/infusion**			Ω	
SPN	60 (50–78)	65 (62–73)	75 (65–75)	65 (57–81)
RPN	85 (60–85)	75 (63–87)	63 (60–75)	70 (60–)
**Glucose, g/infusion**			Ω	
SPN	135 (125–158)	150 (128–175)	160 (130–175)	130 (123–180)
RPN	155 (125–165)	138 (120–175)	136 (120–180)	140 (120–)
**Lipids, g/infusion**	*	*	Ω	γ
SPN	50 (39–58)	55 (51–58)	62 (56 – 75)	65 (59–81)
RPN	60 (56–76)	68 (56–70)	68 (56 – 76)	56 (50–)
**LiMAx, µg/kg/h**		#	*, Ω	
SPN	580 (411–671)	433 (335–532)	439 (287–455)	328 (251–370)
RPN	470 (350–569)	603 (398–786)	610 (515–767)	528 (385–)
**ICG, %/min**				
SPN	25.4 (18.7–29.5)	17.5 (14.9–22.6)	16.0 (13.1–23.2)	16.9 (9.7–20.4)
RPN	20.3 (15.6–23.9)	15.9 (13.4–18.0)	15.7 (12.4–17.3)	17.1 (15.1–)
**FibroScan, kPa**				
SPN	4.9 (4.1–7.1)	5.8 (4.2–10.2)	5.7 (4.3–8.8)	6.8 (4.2–18.2)
RPN	6.8 (5.0–7.8)	5.6 (4.3–10.6)	6.6 (4.3–9.6)	12.5 (5.7–)
**NAFLD Score**				
SPN	−3.62 (−5.34–−1.96)	−2.32 (−4.49–−1.76)	−2.86 (−4.35–−1.18)	−3.24 (−5.21–−0.84)
RPN	−1.74 (−2.91–−0.78)	−1.26 (−2.64–−0.75)	−1.26 (−2.46–−0.74)	−0.69 (−4.01–)
**FIB-4 Score**				
SPN	0.53 (0.35–1.38)	1.15 (0.62–1.49)	0.80 (0.51–2.12)	1.27 (0.58–2.35)
RPN	1.51 (0.72–2.10)	1.86 (1.20–2.15)	1.65 (0.82–2.31)	2.18 (0.56–)
**AST, U/L**				
SPN	30 (21–36)	30 (22–54)	28 (25–40)	29 (22–152)
RPN	37 (24–50)	32 (27–45)	29 (22–32)	19 (26–)
**ALT, U/L**				
SPN	36 (22–65)	31 (24–73)	36 (22–61)	31 (25–149)
RPN	41 (28–104)	30 (19–87)	28 (18–35)	21 (28–)
**AP, U/L**				
SPN	108 (86–378)	93 (73–153)	93 (66–199)	130 (82–188)
RPN	98 (71–150)	101 (74–189)	138 (92–172)	178 (77–)
**GGT, U/L**				
SPN	191 (41–472)	48 (36–135)	61 (38–130)	64 (38–570)
RPN	100 (31–194)	63 (32–120)	63 (37–311)	42 (32–)
**Total Bilirubin, mg/dL**				
SPN	0.44 (0.26–1.00)	0.48 (0.37–1.47)	0.71 (0.24–1.55)	0.91 (0.30–5.1)
RPN	0.39 (0.24–0.44)	0.48 (0.27–0.78)	0.38 (0.26–0.51)	0.38 (0.27–)
**Cholinesterase, kU/L**				
SPN	5.6 (4.0–6.4)	6.2 (4.9–7.6)	5.5 (4.9–7.3)	5.3 (3.4–6.6)
RPN	5.7 (4.0–6.9)	7.5 (5.4–8.9)	5.7 (4.7–6.9)	4.9 (4.9–)
**Citrulline, µmol/L**		#		
SPN	25 (15–41)	28 (17–41)	30 (26–57)	37 (26–65)
RPN	21 (16–23)	24 (22–28)	28 (23–51)	51 (29–)

ALT, alanine aminotransferase; AP, alkaline phosphatase; AST, aspartate aminotransferase; BMI, body mass index; FIB-4, firbrosis-4 score; GGT, gamma-glutamyltransferase; ICG, indocyanine green; LiMAx, maximum liver function capacity; NAFLD, non-alcoholic fatty liver disease; PN, parenteral nutrition; REE, resting energy expenditure; RPN, reduced parenteral nutrition; SPN, stable parenteral nutrition; *, *p* < 0.05 versus comparator group within timepoint; #, *p* < 0.05 vs. previous timepoint; Ω, *p* < 0.05 vs. two timepoints prior; γ, *p* < 0.05 vs. baseline.
